# Eugenic Notes

**Published:** 1914-11

**Authors:** 


### The Department of Eugenics, Mental and Social Hygiene

CONDUCTED BY
DR. MALONE DUGGAN
813-814 Gibbs Building, San Antonio, Texas
All communications for this department should be sent to DR. DUGGAN

### Eugenic Notes.

LANCASTER, PA., has had a cleaning-out of the "social evil," through the efforts of a Law and Order Society, and felicitates herself on the success of the movement in the interest of decency and good government. Who will emulate Lancaster's example for moral reform?

POSITIVE EUGENICS IN NEW YORK.—Governor Glynn, of New York, has appointed a commission of high reputation to investigate the condition of the feeble-minded class of that State, and report to the next Legislature ways and means of coping with the situation. New York is now spending over one-sixth of her revenue in caring for these unfortunates, and they are an open menace to society, not only financially, but by lowering the moral and physical standard and disseminating venereal infection. What about positive eugenics to stop the supply, while philanthropy is humanely condoning for the sins of the fathers?

SOCIAL MORALITY.—Out of much discussion of a subject there must come some good; some chord of real value will be touched that will vibrate through the minds of those that are in tune. Dr. Mabel Ulrich, representing the Commission of Social Morality of the National Board of Young Women's Christian Association, has recently touched some such responsive chords. The doctor takes the position that sex education is needed to strengthen the moral backbone of the individual, and it should be begun early; that the child trained early to learn self-control and appreciation of the esthetic value of his own body and a feeling of responsibility toward the community will react in the right direction when sex temptations come.

The doctor very pertinently called attention to the necessity of knowing the physiology and psychology of sex in women, and the difference in the sex nature of men and women; showing how, with such knowledge, women can be of real assistance to men in their moral tendencies instead of a hindrance; as is so apparent in the dress and manners of women today.

[What society needs most today is real womanly women and real manly men with virility strong but sublimed by love and intellect.]

Of greater significance still, and a point so generally misunderstood, the doctor calls attention to the difference between love and sex passion. She states so truly, "that love must have besides a spiritual and mental value, a physical one, the exclusion of the first two making of the third merely a sex instinct, which in men becomes impersonal, and the person catering to it a sex commodity." She emphasized the individual's increasing feeling of responsibility towards himself and towards society, and the need of woman, if they are to do good work, to work with men and not against them; for only by appreciation of each other's problems in the sex field can a thorough undertsanding and sympathy be developed, and only through such development can the ends aimed for in sex education be attained.

---

A Good Example.—The Medical Association of Maine and the Board of Health of the State concur in the opinion that syphilis, gonorrhea and chancroid should be made reportable diseases, with the understanding that they be reported by number and not by name.

With the view of having legislative enactment on this question, the Society, with the co-operation of the Board of Health, is systematically working up public sentiment in its favor by interesting school superintendents and parents in disseminating information regarding venereal diseases, using "Boys' Venereal Peril," issued by the American Medical Association, as matter of information.

This is a good example for every State to follow. Public sentiment is first necessary to the success of every movement, and the Maine idea is in the right direction. *Who will be next?*

---

The Injunction and Abatement Law in Operation.—The American Social Hygiene Association recently made a field investigation on the effect of the Injunction and Abatement Law as enforced in Iowa and Nebraska. The conclusions of the committee were definite, and may be summarized as follows:

The closing of segregated districts has lessened the evil of prostitution; certainly in quality and probably in quantity. It has done away with trafficking in women for immoral purposes, and the grafting that has so long disgraced so many of our city governments. It has proven conclusively that segregation does not segregate; that the better classes of prostitutes do not live in such districts but always in the residence neighborhoods.

The enforcement of the law has invariably led the owners of houses to abate the nuisances before injunction was filed, and, therefore, there have been no penalties inflicted nor has there been any case of blackmail.

The abolition of the districts has in no instances increased sexual crimes against innocent women. The claim that such districts are necessary to satisfy the animal passion of the predatory male, and, therefore, serve as a moral necessity in the community, has been completely disproved.

The law has influenced unwilling or indifferent public officials to close such districts either by use of this law, or by executive action, and very few private citizens have been compelled to resort to such action. It further permanently and promptly, without evasion or delays of law, cleaned out the business from abuses complained of. The commission found that in no place where the law operated was prostitution open and notorious.—*Abstracts from the A. S. H. S. Bulletin.*

